# Isolation and characterization of ɸEcM-vB1 bacteriophage targeting multidrug-resistant *Escherichia coli*

**DOI:** 10.1186/s13104-024-07033-x

**Published:** 2025-01-03

**Authors:** Rania Abozahra, Dina Shlkamy, Sarah M. Abdelhamid

**Affiliations:** Department of Microbiology and Immunology, Faculty of Pharmacy, Damanhour University, Damanhour, Egypt

**Keywords:** Bacteriophage, Multidrug resistance, *Escherichia coli*

## Abstract

**Objectives:**

The aim of this study is to screen for, isolate and characterize a bacteriophage designated ɸEcM-vB1 with confirmed lytic activity against multidrug-resistant (MDR) *E. coli*. Methods done in this research are bacteriophage isolation, purification, titer determination, bacteriophage morphology, host range determination, bacteriophage latent period and burst size determination, genomic analysis by restriction enzymes, and bacteriophage total protein content determination.

**Results:**

ɸEcM-vB1 bacteriophage exhibited high lytic activity against different MDR *E. coli* isolates and showed stability over wide pH and temperature range. It belongs to the Myoviridae family of the caudovirales order according to TEM. It had a latent period of 5 min and an average burst size of 271.72 pfu/cell. Genomic analysis revealed that it is susceptible to digestion by *EcoRI*. Ten structural proteins were detected by SDS-PAGE. ɸEcM-vB1 is considered a promising candidate for phage therapy applications.

## Introduction

Antimicrobial resistance, which kills 70,000 people annually and is currently the second greatest cause of death globally, is predicted to exceed that of cancer and reach a death rate of 10 million by 2050 [1]. Among those bacteria that are considered the greatest threat to public health are members of the Enterobacteriaceae family, particularly *E. coli*, which is a crucial target in the fight against antibiotic resistance due to its capacity to colonize the guts of both humans and animals, which facilitates organism transmission through the fecal-oral route and its ability to transfer and uptake antibiotic resistance genes via plasmids to and from other bacteria [2]. This necessitates coordinated efforts toward the establishment of alternative solutions to antibiotics as soon as possible. Therefore, bacteriophages are promising candidates that may be used to combat bacterial infections resistant to antibiotics due to their antibacterial features and high specificity [3].

Bacteriophages (phages) are the most prevalent viral entity on Earth and can be found in every ecosystem. Phages multiply within bacterial cells using the cellular machinery after entering the cell, utilizing energy-producing and host protein-synthesizing mechanisms [4]. In this study, a virulent phage (ɸEcM-vB1) that infects multidrug-resistant *E. coli* was isolated from sewage water and characterized.

## Materials and methods

### Bacterial identification and growth conditions

A total of 65 MDR *E. coli* isolates were collected from various clinical samples from Damanhur Medical National Institute Laboratory. The bacterial isolates were identified phenotypically via conventional techniques and confirmed by the VITEK2 system [5]. Strains of *K. pneumoniae*, *A. baumannii*, and *P. aeruginosa* were also collected, identified by the VITEK2 system and used for host range determination.

### Antibiotic susceptibility testing

The sensitivity of the *E. coli* isolates to 15 different antibiotics was evaluated using the Kirby-Bauer disc diffusion method [6] .The inhibition zone diameter of the antibiotic disks was measured, and the results were expressed as either sensitive (S), intermediate (I) or resistant (R) depending on the Clinical and Laboratory Standards Institute (CLSI) 2020 guidelines [7, 8].

### Bacteriophage isolation

Sewage water samples were taken from the Damanhur Medical National Institute and many other locations in Damanhur city, Egypt. Using a spot test, the samples were examined for the presence of phages that may form plaques on MDR *E. coli* isolates [9]. Briefly, sewage samples (15 ml) were centrifuged at 9000 × g for 10 min at 4 °C. The supernatant was then filtered through a 0.45 μm pore size cellulose acetate (CA) membrane filter. Ten milliliters of indicator bacteria were combined with a 5 ml of the sewage filtrate during the exponential development stage. After an overnight incubation at 37 °C, the mixture was centrifuged for 10 min at 12,000×g. Then, the filtered supernatant was checked for the presence of a clear and turbid zone.

### Bacteriophage titer determination

A double-layer agar technique was employed to determine the phage titer and verify the presence of lytic phage. To make double-agar layer (DAL) plates, the filtrate was serially diluted in Luria-Bertani (LB) broth, and then each dilution (100 µl) was combined with 100 µl of *E. coli* suspension. The fresh LB agar plate was overlaid with 4 ml of soft agar (1.2% agar) containing the filtered diluted phage sample and *E. coli*. The plates were then inverted, and incubated overnight at 37 °C and Plates with 30–300 plaques were counted [10].

### Bacteriophage purification and propagation

Bacteriophages were propagated and purified from single-plaque isolates as previously described [11]. Enriched samples may contain more than one phage, and this can be visualized from different sizes and shapes of plaques resulting from agar overlay method. Each plaque was isolated by picking large, clear and non-turbid plaques and resuspended in 1 ml of LB broth. The isolated phages were purified by three successive single-plaque isolations with a sterile Pasteur pipette until homogenous uniform plaques were obtained. The purified phage lysate was kept at 4 °C [9].

### Phage host range determination

We employed 65 clinical isolates of MDR *E. coli*, 5 *K. pneumoniae*, 4 *A. baumannii*, and 2 *P. aeruginosa* for host range determination using spot test [12]. Clear plaques showed a high degree of host specificity, but turbid or no plaques showed non infectivity.

### Examination of phage morphology by transmission electron microscopy

The phage morphological features were investigated using TEM (JEM-1400plus) at the Faculty of Science, Alexandria University, at 80 kV operating voltage. A drop of pure high-titer phage was placed on carbon-coated copper grids (400 mesh). The grids were air-dried for five minutes after being negatively stained with 2% uranyl acetate [11]. The guidelines of the ninth report of the International Committee on Taxonomy of Viruses affirmed the bacteriophage taxonomy and morphology [13].

### Single-step growth curve analysis

The virulent ɸEcM-vB1 latent period and burst size were obtained using a one-step growth experiment, as described previously [14]. Briefly, 10 ml of the host strain combined with purified phage lysate at 0.01 multiplicity of infection (MOI). The mixture was centrifuged for 10 min at 12,000 rpm, and the pellet was resuspended in 10 ml fresh LB broth and incubated at 37 °C. For a total of sixty minutes, 100 µl of the mixture were sampled at intervals of 5 min. The phage titer in the culture was measured by the double agar overlay technique and is expressed as pfu/ml.

### Bacteriophage pH and thermal stability

For pH stability testing, phage suspension (5 × 10^7^ PFU/ml) was exposed to various pH values (1–12, adjusted with either 0.1 M HCl or 0.1 M NaOH) at 30 °C for 16 h of incubation [15]. Phage survival was assessed using both the plaque assay and the spot test.

For thermal stability testing, phage suspension (8 × 10^5^ PFU/ml) was incubated at various temperatures (40–90 °C, adjusted using an incubator) for 20, 40 and 60 min [16]. After incubation, phage titers were measured using a double-layer agar overlay technique.

### Bacteriophage genomic DNA extraction and sensitivity assessment by digestion profile

Phage DNA was obtained using a genomic DNA extraction kit (QiAamp Dsp virus spin kit, QIAGEN). The purified nucleic acid of the phage was visualized on gel electrophoresis and examined for its sensibility versus *EcoRI*,* HindIII*, and *BfaI* enzymes (Fermentas/Thermo Fisher Scientific, USA). UV transilluminator was used to visualize the results using 1% agarose gel [17].

### Bacteriophage proteomics pattern appraisement by sodium dodecyl sulfate‒polyacrylamide gel electrophoresis (SDS‒PAGE)

The purified phage sample was mixed with SDS buffer and added to an 8–12% SDS‒PAGE gel (Bio-Rad). After electrophoresis, Coomassie brilliant blue dye R-250 was used to stain the gel. Molecular size estimation was performed using Novex™ sharp prestained protein standard (Life Technologies). Image acquisition and analysis were performed with Gel Doc XR+ (Bio-Rad) and Image Lab software [18].

### Correlation between antimicrobial resistance and bacteriophage susceptibility

The association between the antimicrobial resistance of all tested *E. coli* isolates (65 isolates) in the study and the antimicrobial resistance of the phage susceptible isolates (33 isolates) was investigated to show which kind of drug-resistance would this phage therapy be effective against and to confirm its use as alternative to antibiotics to treat multidrug resistant bacteria.

## Results

### Identification of *E. coli* isolates and growth conditions

Microbiological testing of the 65 *E. coli* isolates was done by conventional methods and biochemicals tests. Identification was confirmed by Vitek2 system.

### Antibiotic susceptibility testing

The data showed that 7.7 % (5/65) of the *E. coli* isolates were resistant to ertapenem, 9.2 % (6/65) of the isolates were resistant to nitrofurantoin, 21.5% (14/65) of the isolates were resistant to chloramphenicol, 47.7 % (31/65) of the isolates were resistant to tetracycline and gentamicin, 53.8% (35/65) of the isolates were resistant to piperacillin-tazobactam, 67.6% (44/65) of the isolates were resistant to trimethoprim-sulfamethoxazole, 70.7% (46/65) of the isolates were levofloxacin resistant, 81.5% (53/65) of the isolates were cefepime resistant, 95.4% (62/65) of the isolates were cefazoline and ceftriaxone resistant, 97% (63/65) of the isolates were resistant to ciprofloxacin, and finally, ceftazidime, ampicillin, and amoxicillin-clavulanate were not effective against 100% (65/65) of the isolates (Table 1). All 65 isolates were MDR.

The MDR E. coli 3* was selected as an indicator host strain.

**Table 1:**
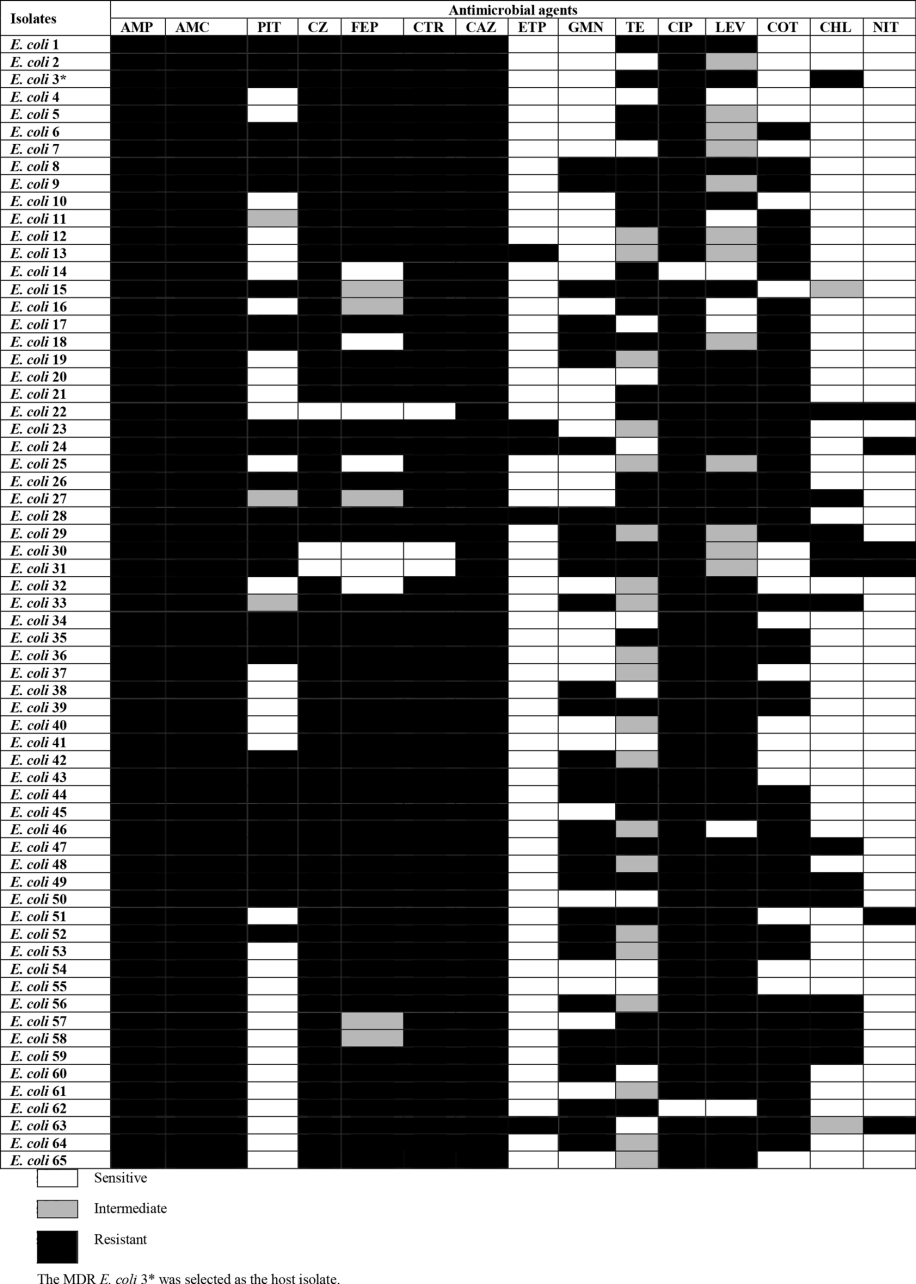
Antibiotic susceptibility of MDR *E. coli* isolates

### Bacteriophage isolation and plaque morphology

**Fig. 1:**
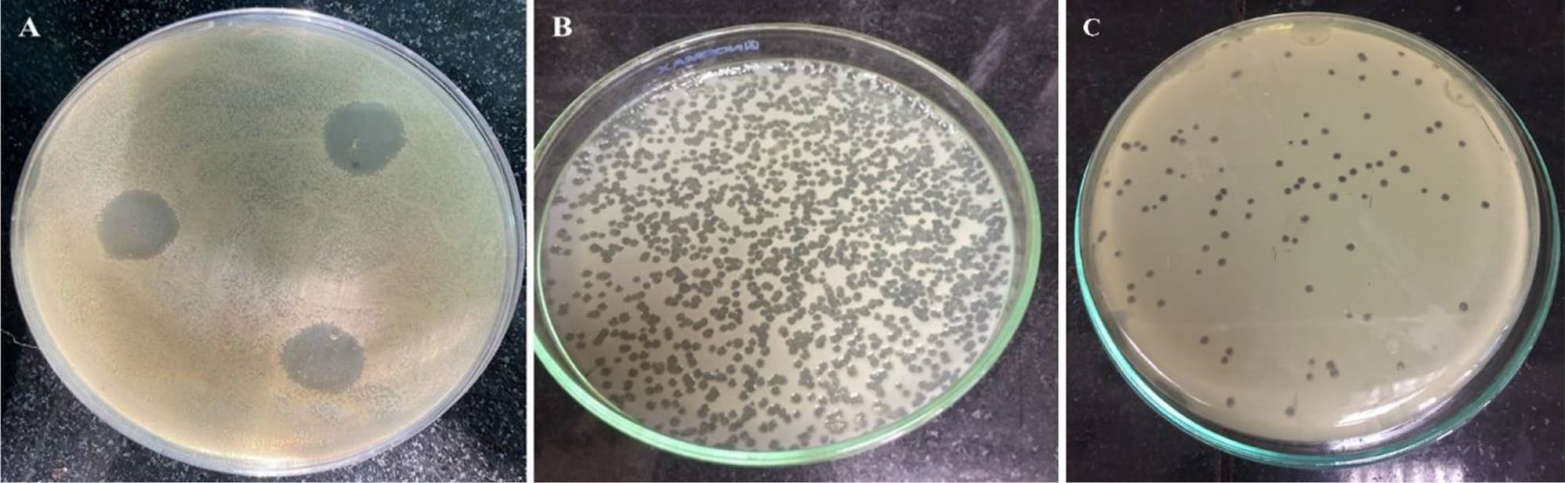
Plaques produced by ɸEcM-vB1 against *E. coli* by using the spot test (**A**) double agar overlay method (**B**) plaques after serial dilution appeared (**C**)

A lytic bacteriophage, designated ɸEcM-vB1 according to a guide for naming and classifying the isolated phage [18], was isolated from sewage water. Our results showed that clear plaques appeared at 37 °C after 18 h of incubation indicating the presence of phage (Fig. 1). After isolation, one plaque was selected for further purification, amplification, and characterization.

### Phage host range determination

The host range of ɸEcM-vB1 was evaluated against 65 MDR *E. coli* isolates and other bacteria (Table 2). ɸEcM-vB1 phage infect 51% (33/65) of the tested *E. coli* isolates. However, it had a minimal effect on other types of bacteria. Only one of the *A. bauminni* isolates was susceptible to our phage.

**Table 2:** Host range of ɸEcM-vB1 bacteriophage

Host	Susceptibility to ɸEcM-vB1		Host	Susceptibility to ɸEcM-vB1
*E. coli 1*	-		*E. coli 39*	-
*E. coli 2*	+		*E. coli 40*	+
E. coli 3*	+		*E. coli 41*	+
*E. coli 4*	+		*E. coli 42*	+
*E. coli 5*	+		*E. coli 43*	+
*E. coli 6*	-		*E. coli 44*	+
*E. coli 7*	-		*E. coli 45*	+
*E. coli 8*	-		*E. coli 46*	+
*E. coli 9*	+		*E. coli 47*	-
*E. coli 10*	+		*E. coli 48*	+
*E. coli 11*	-		*E. coli 49*	-
*E. coli 12*	+		*E. coli 50*	+
*E. coli 13*	-		*E. coli 51*	-
*E. coli 14*	+		*E. coli 52*	+
*E. coli 15*	+		*E. coli 53*	+
*E. coli 16*	-		*E. coli 54*	+
*E. coli 17*	+		*E. coli 55*	+
*E. coli 18*	-		*E. coli 56*	-
*E. coli 19*	-		*E. coli 57*	-
*E. coli 20*	+		*E. coli 58*	-
*E. coli 21*	-		*E. coli 59*	+
*E. coli 22*	+		*E. coli 60*	-
*E. coli 23*	-		*E. coli 61*	+
*E. coli 24*	-		*E. coli 62*	+
*E. coli 25*	+		*E. coli 63*	-
*E. coli 26*	-		*E. coli 64*	+
*E. coli 27*	-		*E. coli 65*	+
*E. coli 28*	-		*K. pneumonia 1*	-
*E. coli 29*	-		*K. pneumonia 2*	-
*E. coli 30*	-		*K. pneumonia 3*	-
*E. coli 31*	-		*K. pneumonia 4*	-
*E. coli 32*	-		*K. pneumonia 5*	-
*E. coli 33*	+		*Acinetobacter 1*	-
*E. coli 34*	-		*Acinetobacter 2*	+
*E. coli 35*	-		*Acinetobacter 3*	-
*E. coli 36*	-		*Acinetobacter 4*	-
*E. coli 37*	+		*Pseudomonas 1*	-
*E. coli 38*	-		*Pseudomonas 2*	-

+: indicates that the strain is susceptible to the phage and that clear plaques were produced

-: indicates that no plaques were observed

### Phage morphology

**Fig. 2:**
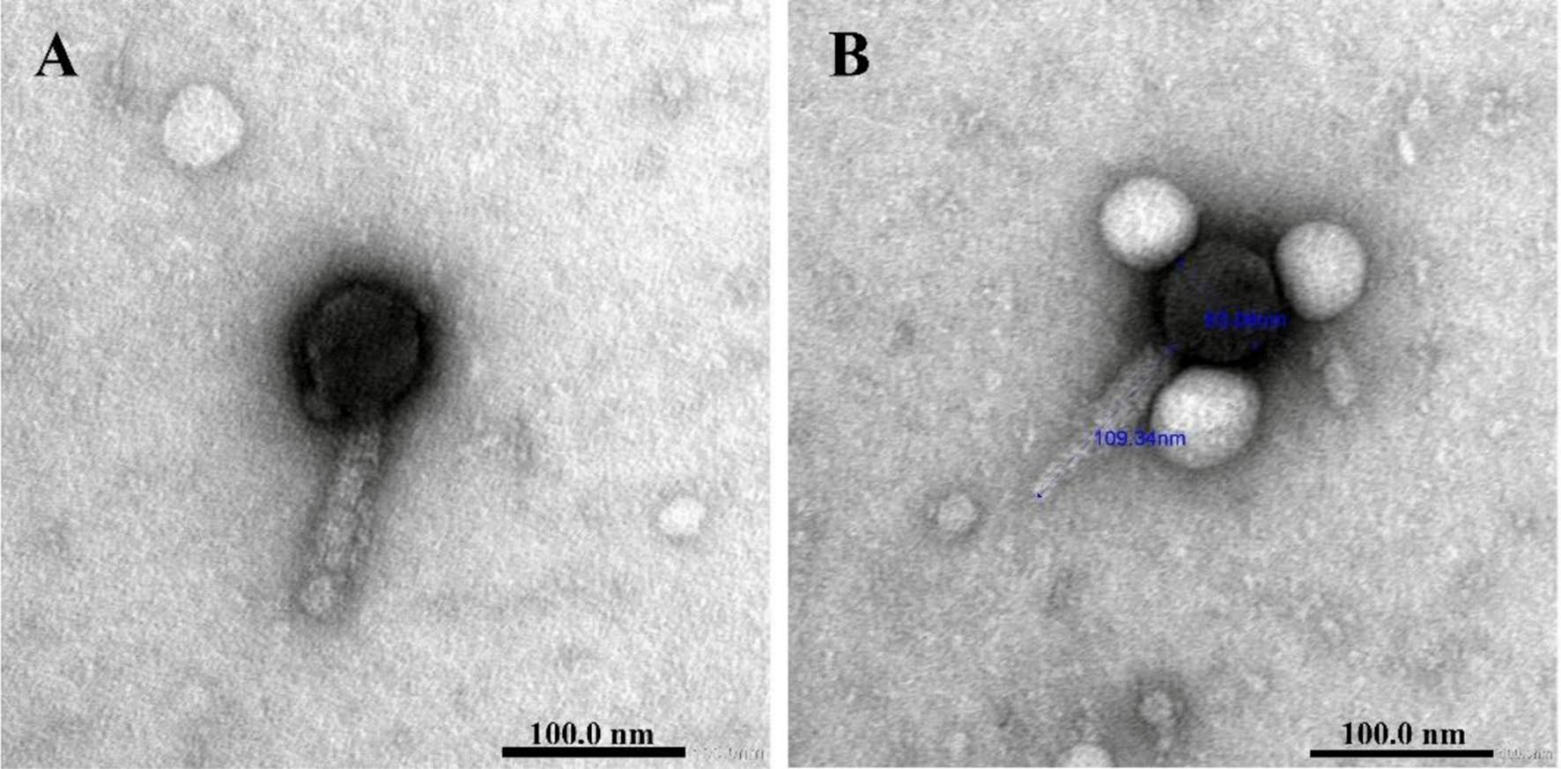
Transmission electron micrographs of purified ɸEcM-vB1 phage

ɸEcM-vB1 had an icosahedral head measuring 63.06 nm in diameter and a long, contractile tail measuring 109.34 nm in length (Fig. 2). It was classified as a member of the caudovirales order and the Myoviridae family.

### Single-step growth curve analysis

**Fig. 3:**
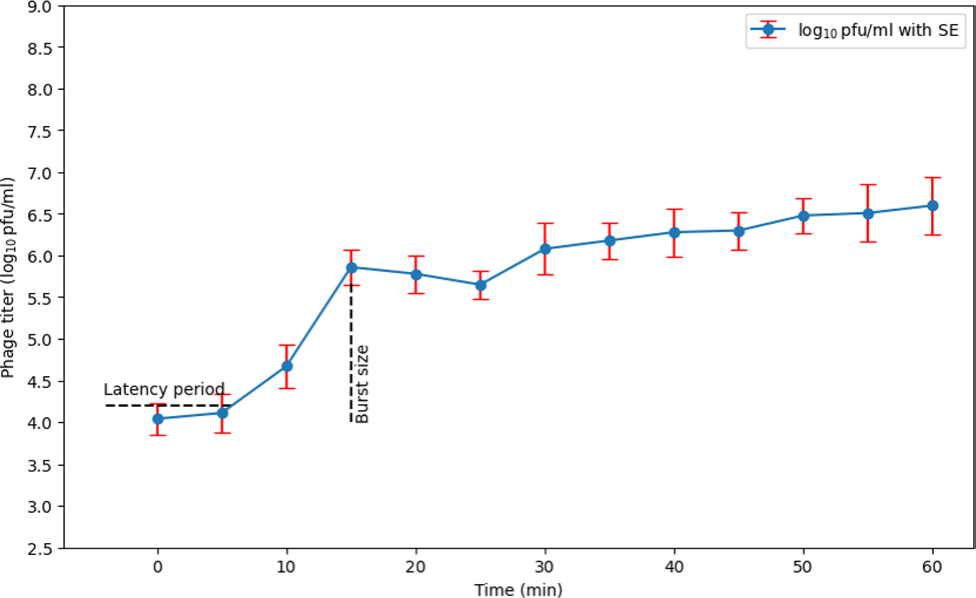
Single-step growth curve of ɸEcM-vB1 using *E. coli* as a host. The results are expressed as the mean ± standard error from three independent experiments

Our results indicated that the latent period of ɸEcM-vB1 phage was 5 min, and the average burst size was 271.72 (Fig. 3).

### Bacteriophage thermal and pH stability

**Fig. 4:**
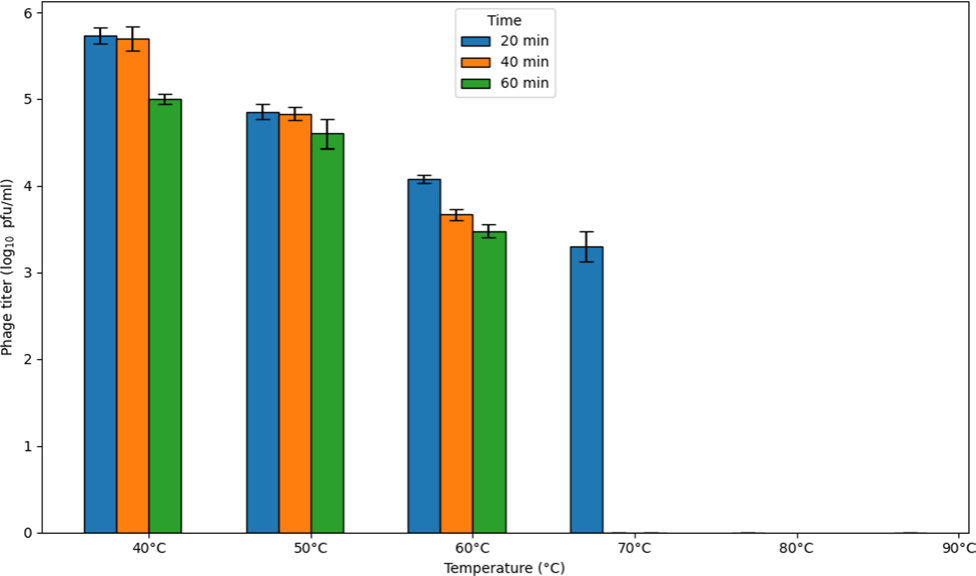
Effect of temperature on the stability of the ɸEcM-vB1 phage. The results are expressed as the mean ± standard error

The results indicated that the virulent phage titer was stable at approximately 5.7 Log_10_ pfu/ml for 40 min at 40 °C. At 50 °C, the phage titer decreased after 20 min to 4.85 Log_10_ pfu/ml. The titer decreased to 3.6 Log_10_ pfu/ml after 40 min at 60 °C, then the phage lost its infectivity after 40 min at 70 °C (Fig. 4).

**Fig. 5:**
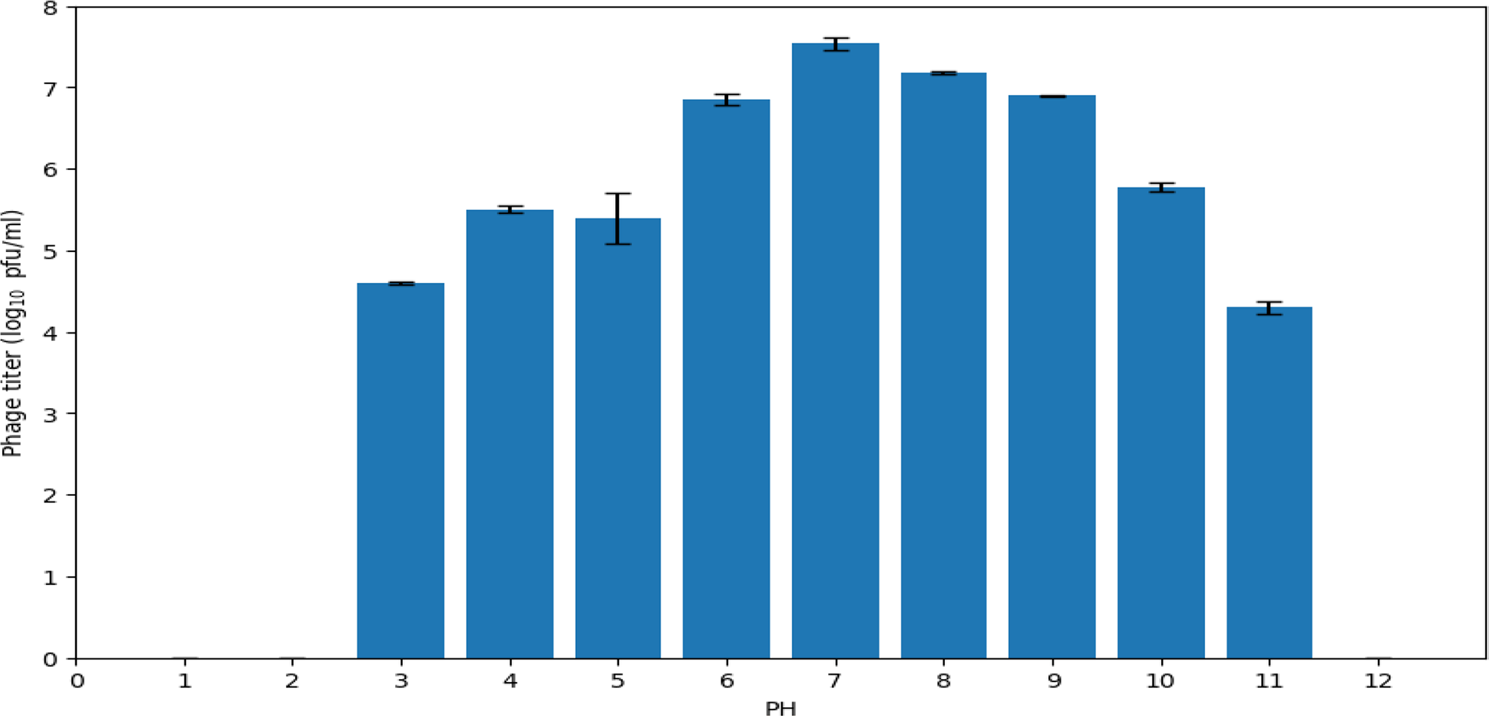
Effect of pH on the stability of the ɸEcM-vB1 phage. The results are shown as the mean ± standard error

The phage was able to survive over a broad pH range (3–11), with peak activity at pH 7, where the titer was 7.5 Log_10_ pfu/ml. Our study revealed greater phage stability at alkaline pH (phage titer is 7.2 Log_10_ pfu/ml at pH 8) compared to acidic pH (phage titer is 6.8 Log_10_ pfu/ml at pH 6). Relatively low titers of our phage, with 4.6 Log_10_ pfu/ml and 4.3 _log10_ pfu/ml, were observed at pH 3 and 11, respectively (Fig. 5).

### Restriction map and protein profile of the isolated bacteriophage

**Fig. 6:**
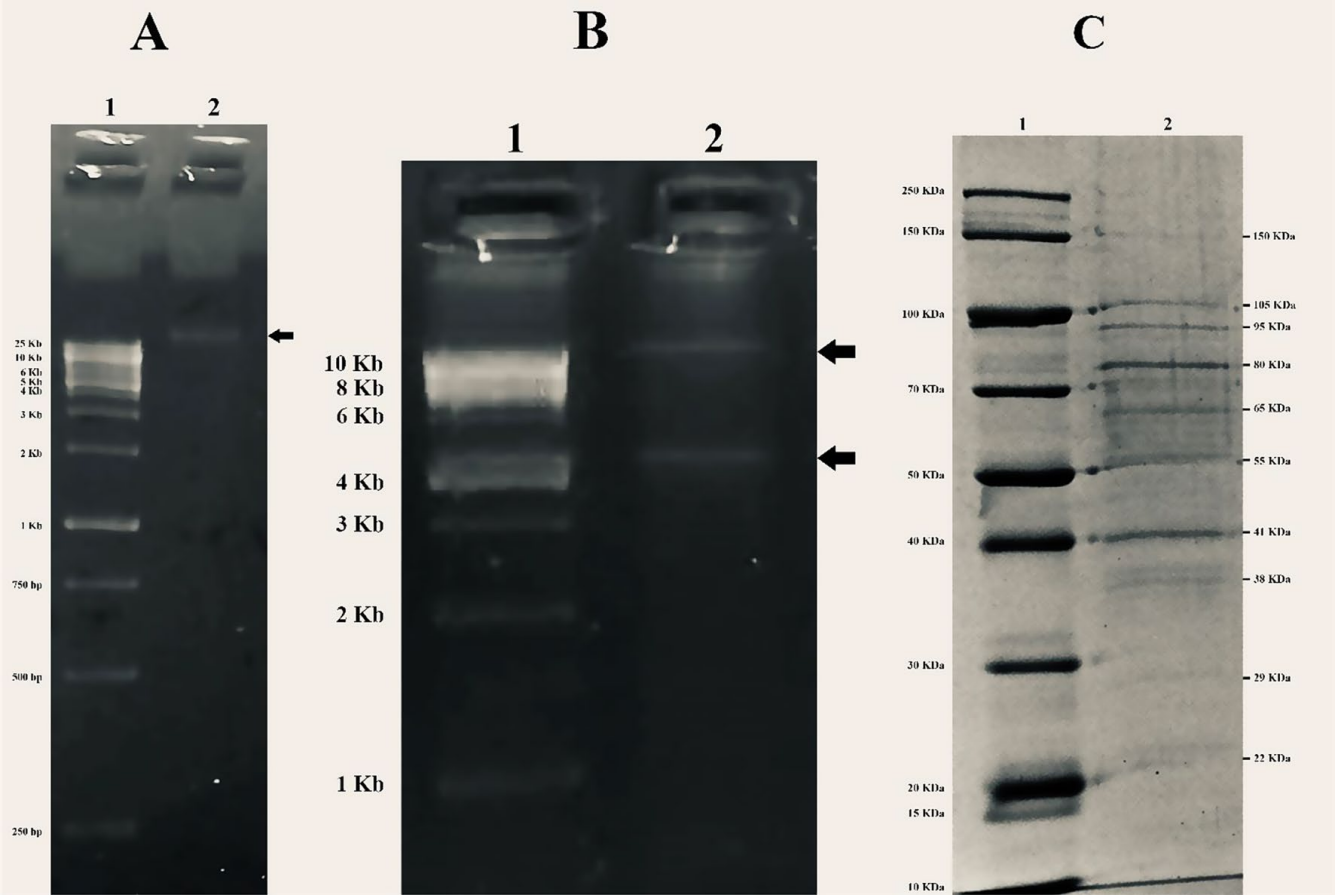
Results of agarose gel electrophoresis and sodium dodecyl sulfate‒polyacrylamide gel electrophoresis; **A** the ɸEcM-vB1 phage genome, as detected by agarose (0.7%) gel electrophoresis. Lane 1 shows the XLarge DNA ladder (Gene DireX) and lane 2 shows a band of phage DNA of size more than 25 kb, **B** Lane 1 shows a 1 kb DNA ladder (New England Biolabs) and lane 2 shows the ɸEcM-vB1 phage DNA restriction analysis with EcoR1; **C** Image shows the SDS‒PAGE analysis of the ɸEcM-vB1 phage structural proteins; lane 1 shows broad range protein molecular weight markers (The Novex™ sharp pre-stained protein standard, Life Technologies) and lane 2 shows the ɸEcM-vB1 phage proteins

Our results showed that the genomic DNA of size more than 25 kb (Fig. 6A), was digested by *EcoRI* producing two digest pattern fragments (Fig. 6B). *HindIII* and *BfaI* had no effect on the phage genome. It was estimated that the phage ɸEcM-vB1 had 10 structural proteins with sizes ranging from 22 to 150 kDa (Fig. 6C).

### Correlation between antimicrobial resistance and bacteriophage susceptibility

**Fig. 7:**
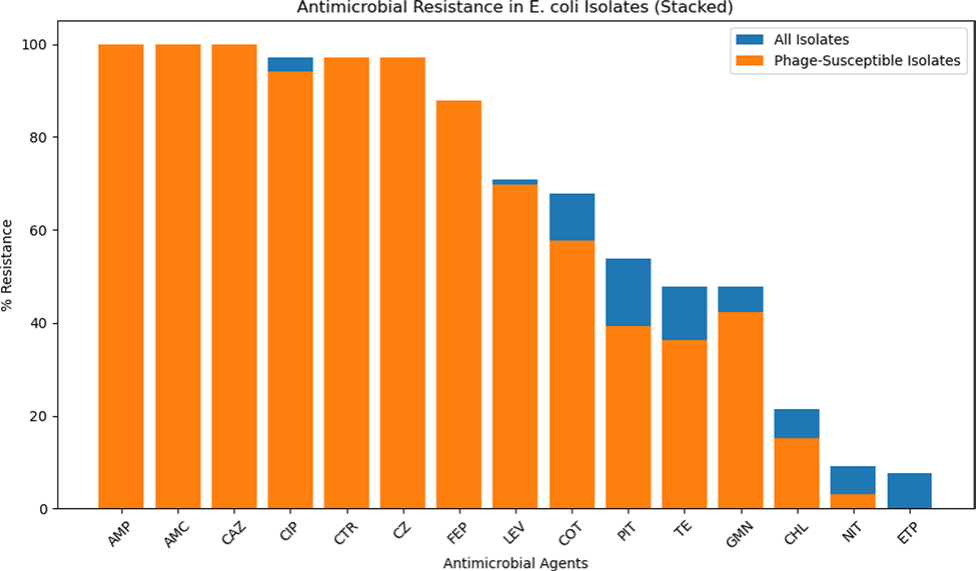
Correlation analysis of antimicrobial resistance and bacteriophage treatment

Our results indicated an extremely strong positive correlation, suggesting that bacteriophage therapy may be broadly effective across different MDR *E. coli* strains. Our phage showed sensitivity to isolates that resist all antimicrobial agents used in the study with different percentage except for Ertapenem antibiotic as shown in Fig. 7.

### Statistical analysis

The experiments were done in triplicates, and the data were expressed as mean ± standard deviation (SD). The data were then analyzed using Python libraries (numpy – pandas – matplotlib – SciPy) to plot effect of time, temperature, and PH on phage titer measures. Shapiro test was done to see the normality of distribution of % resistance data which were found to be not normally distributed. Spearman correlation test was done to find the relation between (% resistance in all isolates) against (% resistance in phage susceptible isolates) where the significance level was set at *p* ≤ 0.05.

## Discussion

In this study, a lytic bacteriophage, designated ɸEcM-vB1, was successfully isolated and identified from hospital sewage samples. According to its TEM image, it resembles the typical structural features of previous reported phages [19–21]. It was classified as a member of the *Myoviridae* family, *caudovirales* order, which is considered the preferred therapy [22]. Moreover, Myoviridae phages are considered the most promising because they are classified as virulent and cannot mobilize or transfer genetic information [23]. The clear plaques indicated that the available phage was virulent. The advantage of our isolated phage is that it shows strong lytic activity against a variety of MDR *E. coli* isolates with a coverage of 51% of the tested *E. coli* isolates, which is similar to reported phages [19, 24, 25] and in contrast with other studies where phages had a limited host range [26, 27]. ɸEcM-vB1 had a minimal effect on other types of bacteria, as it had no lytic effect on *K. pneumoniae* and *P. aeruginosa* isolates. The phage infects one of the Acinetobacter strains which is interesting because acinetobacter phages are usually unstable. The limited host range of phages that selectively target Acinetobacter spp., usually one host one phage, is most likely caused by the bacterium’s abundance of surface bacterial antigens. These antigens are adequate for recognizing distinct phages [28]. This may happen because spot testing technique can sometimes cause false positives because of lysis of bacterial cells without phage infection [29].

Our isolated phage had a short latent time of 5 min and a large burst size of 271.72 phages/infected cell which is similar to previous reported *E. coli* phage, indicating their potential efficacy in phage therapy [27]. Another study revealed that an *E. coli* phage had a 20 min latent period and a burst size of 1200 pfu per infected host [21].

Many studies have documented that bacteriophages may vary in their pH and thermal tolerance [30]. In the current research, isolated phage remained highly viable under physiological conditions in the pH range of 3.0–11.0, which is consistent with other findings [25, 31]. However, in a different investigation, the VB_EcoS-Golestan phage titer was only stable and active at pH values between 7.0 and 8.0 [32]. Moreover, the ɸEcM-vB1 phage also showed great thermal stability between 30 °C and 70 °C. Previous studies revealed that a rise in temperature reduces the phage titer [33]. The isolated phage has higher thermal stability than the previously reported phages PA13076 and PC2184, which only exhibited peak activity between 30 and 50 °C [34].

Phage genomic DNA restriction digestion analysis is one of the easiest and least expensive molecular methods [35]. Our results showed that the ɸEcM-vB1 genomic DNA was digested mainly by *EcoRI.* However, our phage cannot be digested by *HindIII* and *BfaI*, which is similar to the previously reported phages CBA120 and FEC14 [36, 37].

When identifying viruses, molecular techniques such as SDS‒PAGE can be used to detect individual protein molecules since they can quantify the molecular weights of phage proteins [38]. Our results revealed that the phage ɸEcM-vB1 had 10 structural proteins with sizes ranging from 22 to 150 kDa. According to a prior study, the isolated phage’s φEf11 SDS-PAGE examination revealed 11 protein bands with sizes ranging from 27 to 85 kDa [39]. A different investigation showed that eleven proteins with molecular weights ranging from 17 to 200 kDa, were identified with the MJ1 phage [27].

Our findings suggest extremely strong positive correlation between antimicrobial resistance and bacteriophage susceptibility and highlight its potential as a complementary treatment option alongside antibiotics.

## Conclusion

The virulent ɸEcM-vB1 phage can be considered a promising option for application in phage therapy.

### Limitations

A limitation of this study may be whole genome sequencing of ɸEcM-vB1 phage because it is not nationally available at the moment.

## Data Availability

Data is provided within the manuscript or supplementary information files.

## Abbreviations

CA: Cellulose acetate
CLSI: Clinical and Laboratory Standards Institute
DAL: Double-agar layer
LB: Luria-Bertani
MDR: Multidrug-resistant
MOI: Multiplicity of infection
TEM: Transmission electron microscopy
